# Sebaceoma on the nose mimicking basal cell carcinoma: Pitfalls of dermoscopy and reflectance confocal microscopy

**DOI:** 10.1111/srt.13227

**Published:** 2022-11-02

**Authors:** Xiaoli Ning, Huijing Wang, Zhancai Zheng, Ying Wang, Yong Cui

**Affiliations:** ^1^ Peking University China‐Japan Friendship School of Clinical Medicine Beijing China; ^2^ Department of Dermatology China‐Japan Friendship Hospital Beijing China; ^3^ Graduate School Beijing University of Chinese Medicine Beijing China

**Keywords:** basal cell carcinoma, confocal microscopy, dermoscopy, sebaceoma

## Abstract

Sebaceoma is a rare benign sebaceous tumor that usually occurs on the face and scalp. We report a case of a 3‐mm solitary pink papule on the nose in an elderly woman. Dermoscopic examination showed yellow‐pinkish background with a central yellow homogeneous structure, peripheral branching vessels (crown vessels), and scattered gray or reddish‐brown irregular areas. Reflectance confocal microscopy (RCM) revealed tumor islands with massive dendritic cells and scattered bright fine granules in the dermis, a suspicious palisading arrangement at the periphery, and there seemed to be peritumoral dark spaces. The combined dermoscopic and RCM examination were highly suspicious for the diagnosis of basal cell carcinoma (BCC), so the lesion was excised completely, but was eventually diagnosed as sebaceoma by histopathology. This case suggests that there are some overlaps in both dermoscopic and RCM features between sebaceoma and BCC. The application of dermoscopy and RCM to the diagnosis of sebaceoma is challenging, further studies are needed in this field.

Dear Editors,

Sebaceoma is a rare benign sebaceous tumor and was first coined in 1984.^1^ Clinically, it presents as an isolated papule or nodule, less than 1 cm, with a yellow, flesh, or pink color, which is easily confused with basal cell carcinoma (BCC), sebaceous adenoma, and sebaceous carcinoma.^1^ Although the dermoscopic and reflectance confocal microscopic (RCM) patterns of sebaceous tumors have been summarized, to date very little is known about that of sebaceoma. Here, we report a case misdiagnosed as BCC by dermoscopy and RCM, which was eventually diagnosed as sebaceoma by histopathology.

A 76‐year‐old woman presented with a 1‐year history of a pink papule on her nose, which appeared slightly painful and had gradually enlarged over the last 5 months. The patient had pricked the lesion with a needle before the visit, with no prior history of local trauma or infection nor a family history. No correlation was detected with Muir‐Torre syndrome. Physical examination showed a 3‐mm solitary pink papule with a central umbilication and several small black spots on the nose (Figure 1A).

**FIGURE 1:**
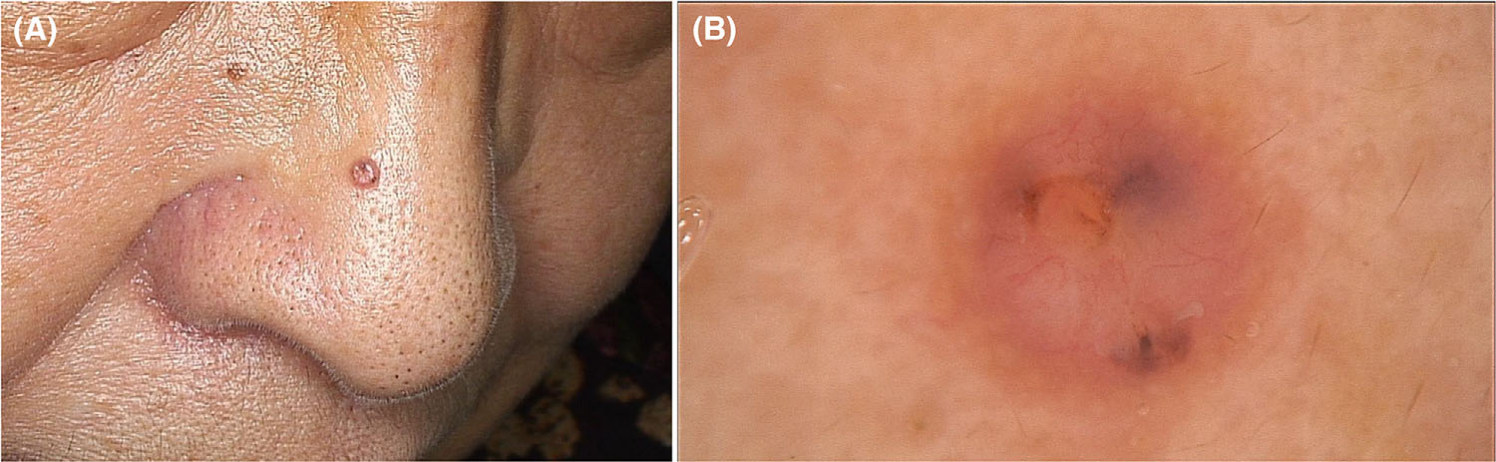
Clinical and dermoscopic manifestations. (A) A 3‐mm solitary pink papule on the , with a central umbilication and several small black spots. (B) The yellow‐pinkish background, a central yellow homogeneous structure, peripheral branching vessels (crown vessels), and scattered gray or reddish‐brown irregular areas on dermoscopy (×50)

Dermoscopic examination demonstrated the yellow‐pinkish homogeneous background with a central yellow homogeneous structure (suspicious ulcer), peripheral branching vessels radiating toward the center (crown vessels), and scattered gray or reddish‐brown irregular areas (Figure 1B). The features of dermoscopy were suggestive of BCC or sebaceous tumor, so RCM examination was further performed, which revealed that roughly regular structures in the epidermis, the epithelial‐connective tissue junction was partially indistinct, presenting numerous high refractive dendritic cells and linear, oval, or branching blood vessels. In the dermis, there were multiple well‐defined tumor islands, where numerous dendritic cells and scattered bright fine granules were visible, as well as peripheral palisading arrangement. The scattered infiltration of inflammatory cells and many dilated blood vessels were also observed. In addition, there seemed to be peritumoral dark spaces (Figure 2).

**FIGURE 2:**
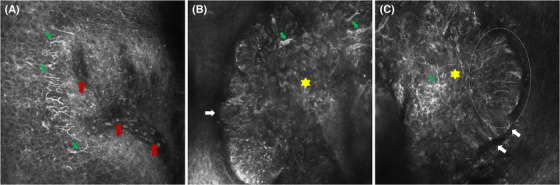
Reflectance confocal microscopic findings. (A) Dark branching blood vessels (red arrows) and bright dendritic cells (green arrows). (B–C) The tumor islands (yellow hexagrams) with numerous dendritic cells (green arrows) and scattered bright fine granules, peripheral palisading arrangement (white circle), dark spaces between islands and stroma (white arrows)

The combination of dermoscopy and RCM were highly suspicious of the diagnosis of BCC, so the lesion was eventually excised completely. Histopathology showed a well‐circumscribed tumor nodule in the dermis, adjacent to the overlying epidermis, surrounded by fibrous tissue and infiltrating lymphocytes. The cystic cavities were visible. The tumor consisted mainly of basaloid cells with round or elongated basophilic nuclei. No pleomorphism, atypical mitoses, or necrosis were identified. There were a few scattered mature sebocytes with scalloped nuclei and vacuolated cytoplasm. The peripheral palisading arrangement and peritumoral clefts were not seen (Figure 3).

**FIGURE 3:**
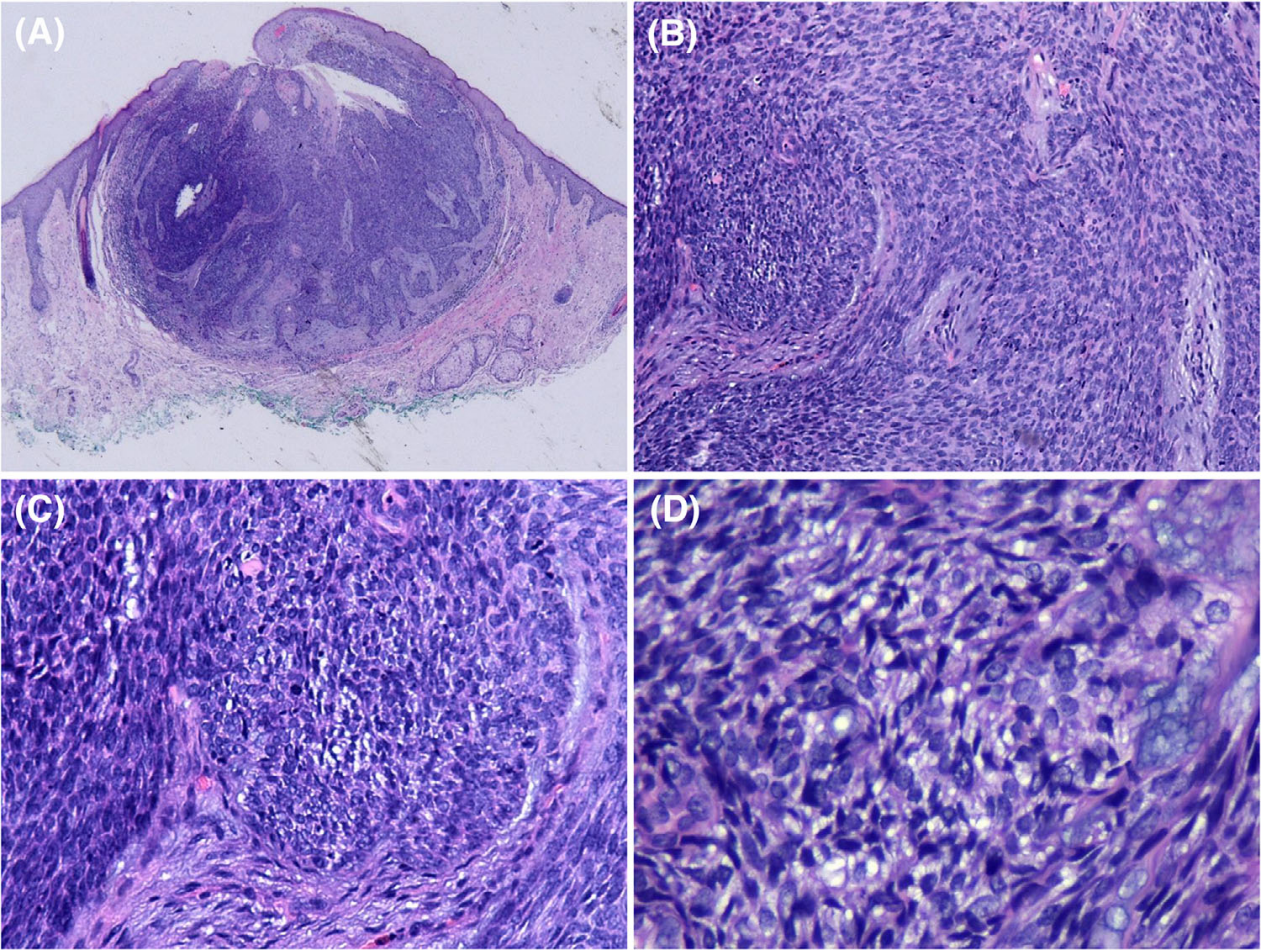
Histopathological features. (A) A well‐circumscribed tumor in the dermis, surrounded by fibrous tissue and lymphocytes (hematoxylin‐eosin, low magnification). (B) Many basaloid cells with round or slightly elongated basophilic nuclei (hematoxylin‐eosin, ×100). (C) No peripheral palisading arrangement and retraction spaces between the tumor lobules and the stroma (hematoxylin‐eosin, ×200). (D) Scattered sebocytes with scalloped nuclei and vacuolated cytoplasm (hematoxylin‐eosin, ×400)

The final diagnosis of sebaceoma was confirmed. No recurrence was detected at the one‐month follow‐up.

The main dermoscopic features of sebaceoma include a white to yellow background, a central crater filled with a yellow homogeneous structure or occasionally with blood crust, and crown or peripherally thin arborizing vessels.^2^, ^3^, ^4^ Unusually, we also observed the gray or reddish‐brown irregular areas, which could easily be misidentified as the blue‐gray globules of BCC. We thought that it was most likely due to subcutaneous hemorrhage caused by the patient's needling, given that no aggregated melanocytes or melanophages were visible in the histopathology.

Sebaceoma is sometimes indistinguishable from BCC on dermoscopy. However, the combination of yellow structures and less bright red crown vessels suggests a sebaceoma, while the bright red, sharply focused arborizing vessels and blue‐grey dots/globules/nests are clues to the diagnosis of BCC, and BCC also lacks a yellow‐white background.^4^, ^5^

In our case, the RCM features of sebaceoma satisfied the three criteria for diagnosing BCC with a specificity of 78%.^6^ Rosales et al. reported that sebaceomas lack space between the tumor and the stroma.^7^ However we observed this structure, only the dark space was narrower than in BCC. The presence of sebocytes with central dark nuclei and abundant cytoplasm filled with bright fine granules is a crucial clue to differentiate sebaceoma from BCC.^6^, ^7^ Surprisingly, no obvious sebocytes were found under RCM in our case. IN our experience, the refractive index of sebocytes is not as high as that of dendritic cells on RCM, and sebocytes only account for a small proportion of the tumor, these lead to difficulties to visualize sebocytes when numerous dendritic cells present within the tumor. And there are some other clues to differentiate BCC from sebaceous tumors on RCM, the structures of streaming and peripheral palisading of nuclei are more prominent in BCC, and the presence of epidermal pleomorphism and large, highly refractive phagocytes are also suggestive of BCC.^6^, ^8^

Interestingly, the peripheral palisading of nuclei was not observed in the histopathology of this sebaceoma, which suggested that the bright radial fine streaks at the periphery of tumor islands under RCM do not necessarily correspond to the palisading nuclei of basaloid cells in histopathology.

To conclude, we report a case of sebaceoma mimicking BCC under dermoscopy and RCM. The application of dermoscopy and RCM to the diagnosis of sebaceoma remained challenging, and more research was needed in this field.

## CONFLICT OF INTEREST

None.

## ETHICS STATEMENT

The written informed consent was signed by the patient.

## Data Availability

The data that support the findings of this study are available from the corresponding author upon reasonable request.
